# Supplementary material for: NSD1 supports cell growth and regulates autophagy in HPV-negative head and neck squamous cell carcinoma
Source: Cell Death Discov. 2024 Feb 13;10:75. doi: 10.1038/s41420-024-01842-6 (PMC10861597; doi:10.1038/s41420-024-01842-6)
Supplement: Supplementary file 1 — Supplementary Figure legends [file 41420_2024_1842_MOESM1_ESM.docx]

**­­­­Supplementary Figure S1**

(A) Relative mRNA level of *NSD1* gene, measured by RT-qPCR in human HNSCC cell lines. *NSD1* relative level was normalized on *18S* as a control gene. Statistical significance was determined by Kruskal-Wallis with Dunn's multiple comparisons post-test.

(B) Relative mRNA level of *NSD2* and *NSD3* genes was measured by RT-qPCR in head and cancer cell lines. *NSD2* and *NSD3* relative level were normalized to *18S* as a control gene.

(С) Western blot of NSD2 and NSD3 protein levels in a panel of human HNSCC cell lines.

(D) Quantification of Western blot images in (C).

(E) mRNA level of *NSD2* and *NSD3* genes was measured by RT-qPCR after NSD1 shRNA knockdown cells at 72h after knockdown induction. *NSD2* and *NSD3* relative level were normalized to *18S* as a control gene. Statistical significance determined by ANOVA with Dunnett multiple comparison post-test.

(F) Western blot of NSD2 and NSD3 protein levels upon induction of pLKO control or NSD1 shRNA knockdown at 72h with doxycycline

(G) Quantification of Western blot images in (F). Statistical significance was determined by ANOVA with Dunnett multiple comparison post-test.

Experiments were performed in at least three independent biological repeats. The error bars are presented as mean ± SEM. ns – not significant, *p<0.05, **p<0.01, ***p<0.001, and ****p<0.0001.

**Supplementary Figure S2**

(A) Representative images of colony formation assay and quantification of the relative colony numbers in a panel of human HNSCC cell lines. Statistical significance was determined by Kruskal-Wallis with Dunn's multiple comparisons post-test.

(B) Representative flow cytometry images (left) and average calculations (right) of the Annexin V/Propidium iodide (PI) staining as measured by flow cytometry at 144h after NSD1 knockdown induction in JHU 011 and Cal27 cell lines. Statistical significance was determined by Kruskal-Wallis with Dunn's multiple comparisons post-test.

(C) Western blot of NSD1 and H3K36me^2^ levels in mice tumors. Mouse number #1 from the control group died on day 23, and the tumor of this mouse was not validated by western blot.

(D) Quantification of Western blot images in (C). Statistical significance was determined by Mann-Whitney test.

Experiments were performed in at least three independent biological repeats. The error bars are presented as mean ± SEM. ns – not significant, *p<0.05, **p<0.01, ***p<0.001, and ****p<0.0001.

**Supplementary Figure S3**

(A) Heat maps of the changed genes from for the gene pathways from (Figure 2B).

(B) Heat maps of the AMPK subunits expression in RNA-sequencing data for JHU 011 and Cal27 cell lines. *PRKAA1* - protein kinase AMP-activated catalytic subunit alpha 1; *PRKAA2* - protein kinase AMP-activated catalytic subunit alpha 2; PRKAB*1 -* protein kinase AMP-activated non-catalytic subunit beta 1; *PRKAB2 -* protein kinase AMP-activated non-catalytic subunit beta 2; *PRKAG1* - protein kinase AMP-activated non-catalytic subunit gamma 1; *PRKAG2* - protein kinase AMP-activated non-catalytic subunit gamma 2; *PRKAG3* - protein kinase AMP-activated non-catalytic subunit gamma 2.

**Supplementary Figure S4**

Quantification of Western blot images in Figure 3C. Statistical significance was determined by Kruskal-Wallis with Dunn's multiple comparisons post-test.

Experiments were performed in at least three independent biological repeats. The error bars are presented as mean ± SEM. ns – not significant, *p<0.05, **p<0.01, ***p<0.001, and ****p<0.0001.

**Supplementary Figure S5**

(A) Quantification of Western blot images in Figure 3D. Statistical significance was determined by Kruskal-Wallis with Dunn's multiple comparisons post-test.

(B) Western blot of pT202/204 MAPK and p44/p42 MAPK total protein levels in NSD1 shRNA knockdown cells at 72h after knockdown induction.

(C) Quantification of western blot images in (B). Statistical significance was determined by Kruskal-Wallis with Dunn's multiple comparisons post-test.

Experiments were performed in at least three independent biological repeats. The error bars are presented as mean ± SEM. ns – not significant, *p<0.05, **p<0.01, ***p<0.001, and ****p<0.0001.

**Supplementary Figure S6**

(A) Western blot of ULK1, p62, and LC3B-II protein levels in ULK1 siRNA knockdown cells at 72h after knockdown induction.

(B) Quantification of Western blot images in (A). Statistical significance was determined by Kruskal-Wallis with Dunn's multiple comparisons post-test.

The error bars are presented as mean ± SEM. ns – not significant, *p<0.05, **p<0.01, ***p<0.001, and ****p<0.0001.

(C) Proliferation of JHU 011 and Cal27 cell lines with siRNA ULK1 knockdown, as measured by CTB assay for up to 168 hours, at indicated time points. Statistical significance was determined by ANOVA with Dunnett multiple comparison post-test. Each group was compared to NC-transfected cells.

**Supplementary Figure S7**

(A) Western blot of assessing mTORC1 signaling pathway and autophagy markers levels upon mTOR inhibition with Rapamycin and chloroquine (CQ).

(B) Quantification of Western blot in (A). Statistical significance determined by ANOVA with Tukey multiple comparison post-test.

The error bars are presented as mean ± SEM. ns – not significant, *p<0.05, **p<0.01, ***p<0.001, and ****p<0.0001.
