# Supplementary material for: NSD1 supports cell growth and regulates autophagy in HPV-negative head and neck squamous cell carcinoma
Source: Cell Death Discov. 2024 Feb 13;10:75. doi: 10.1038/s41420-024-01842-6 (PMC10861597; doi:10.1038/s41420-024-01842-6)

Supplementary Figure S1

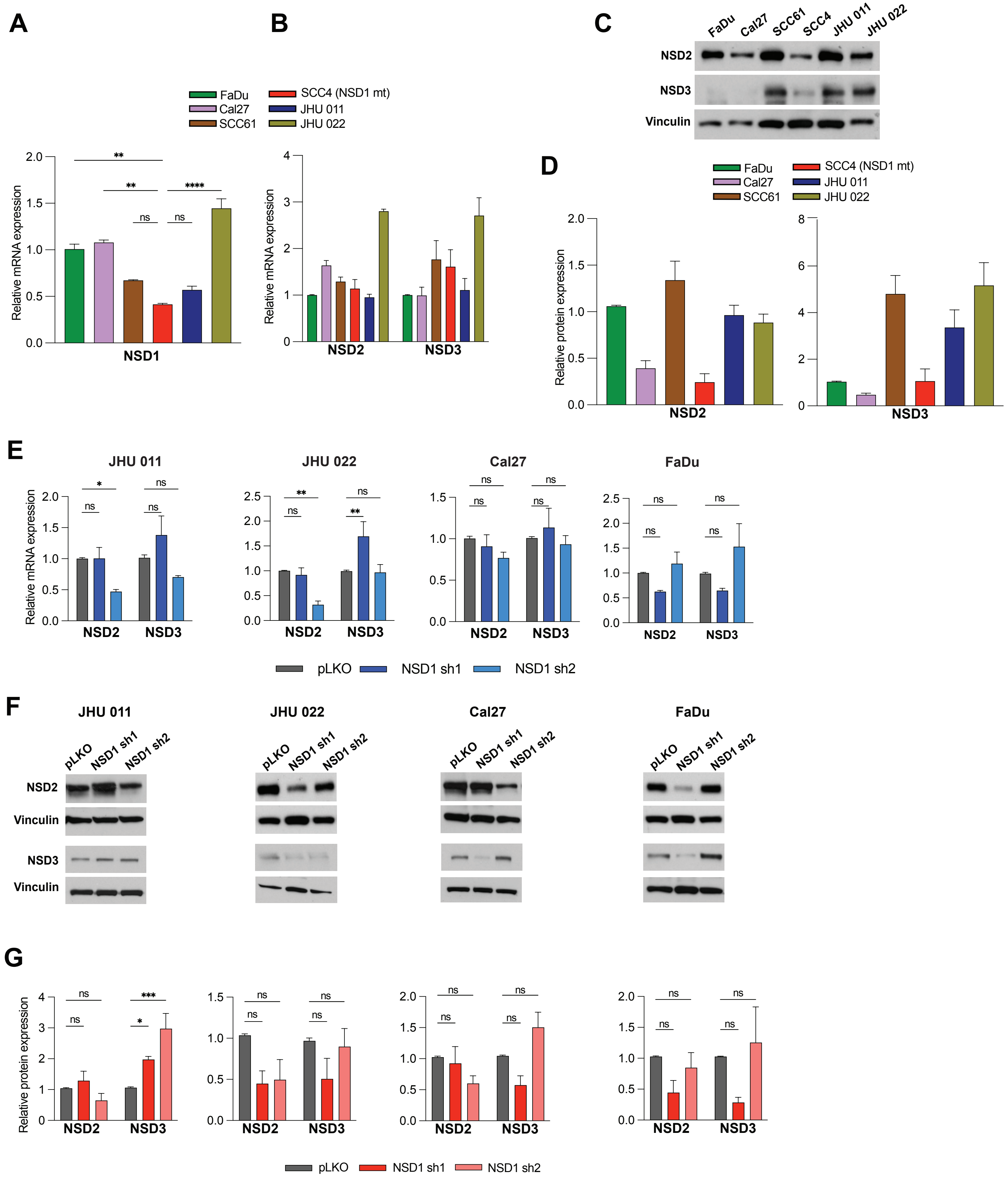

Supplementary Figure S2

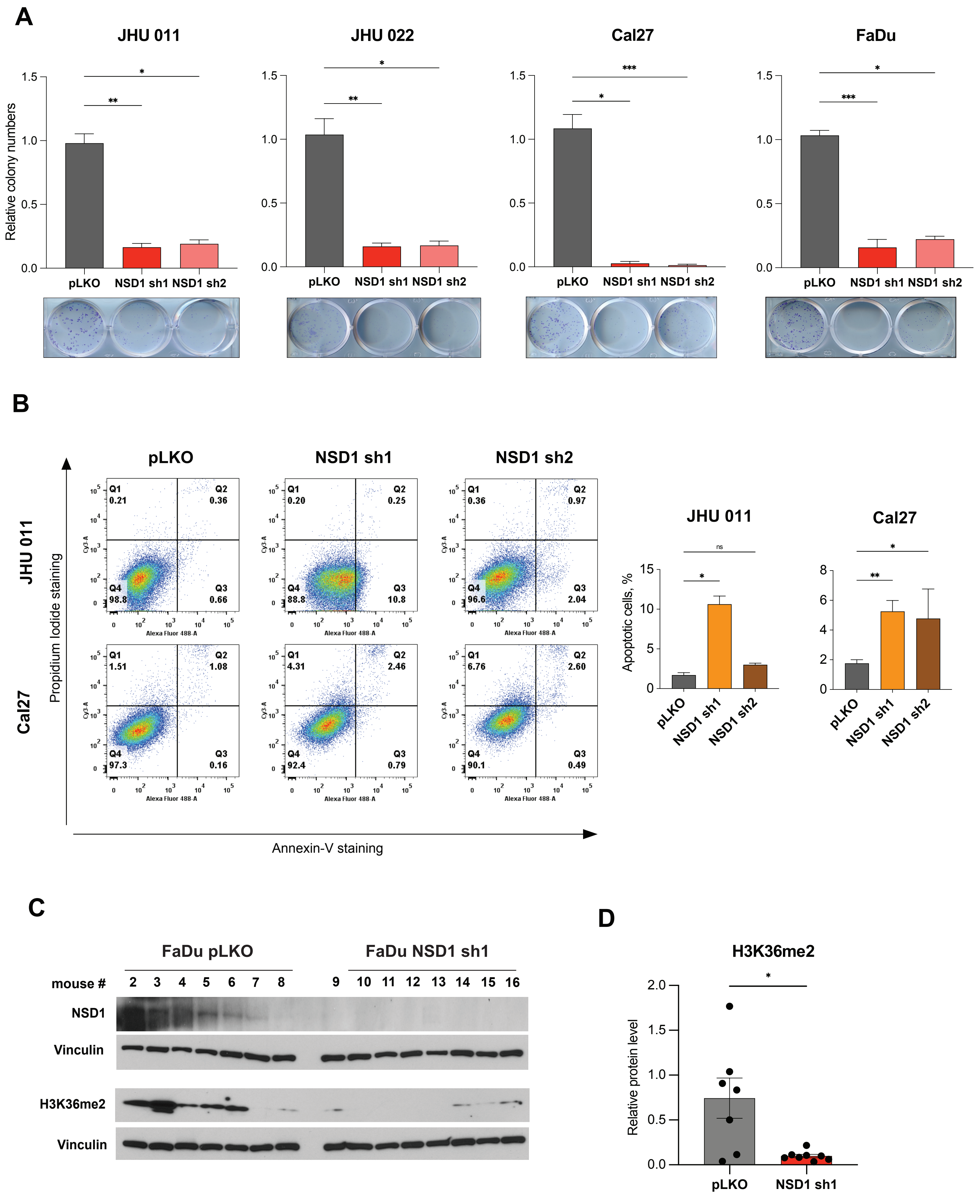

Supplementary Figure S3

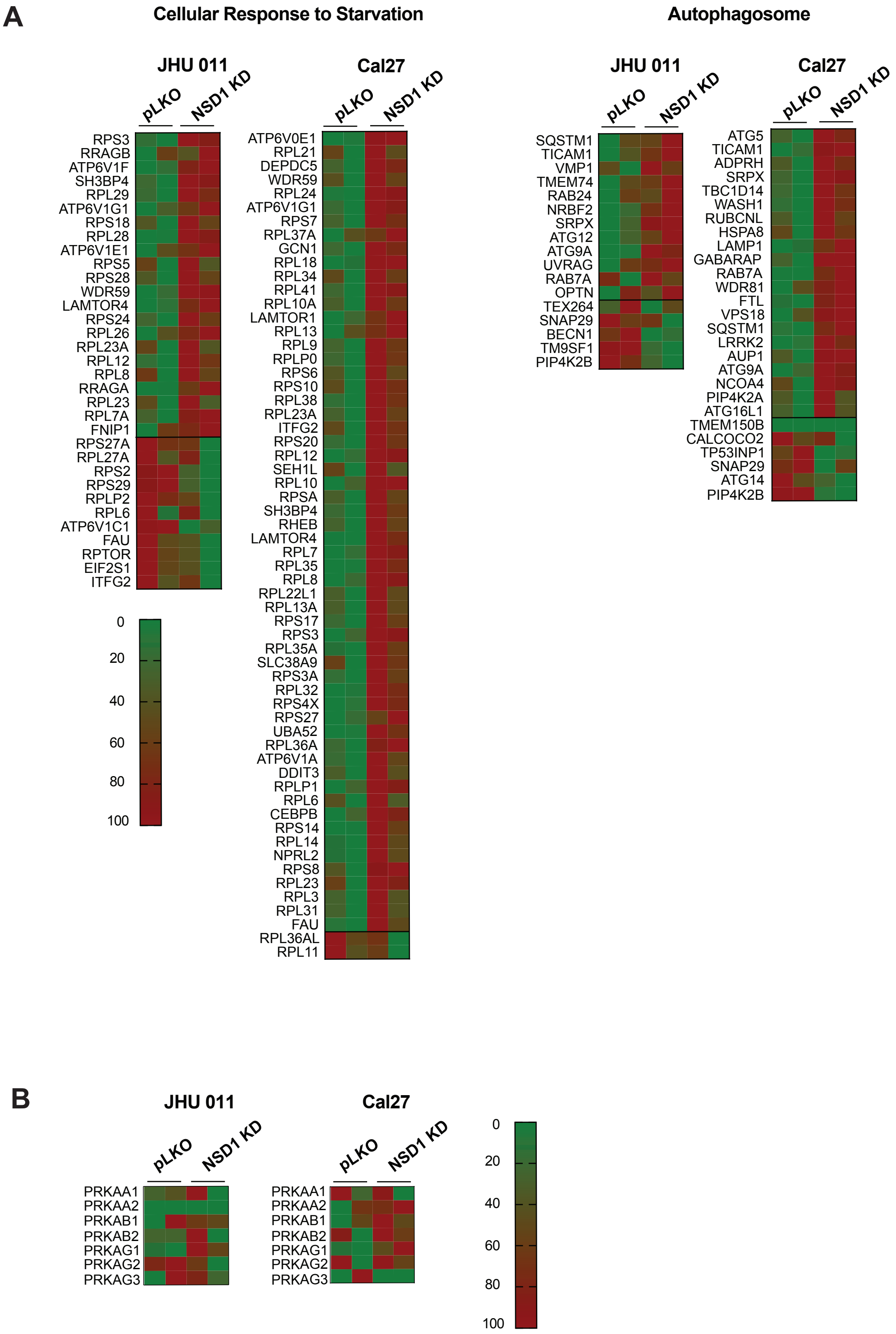

Supplementary Figure S4

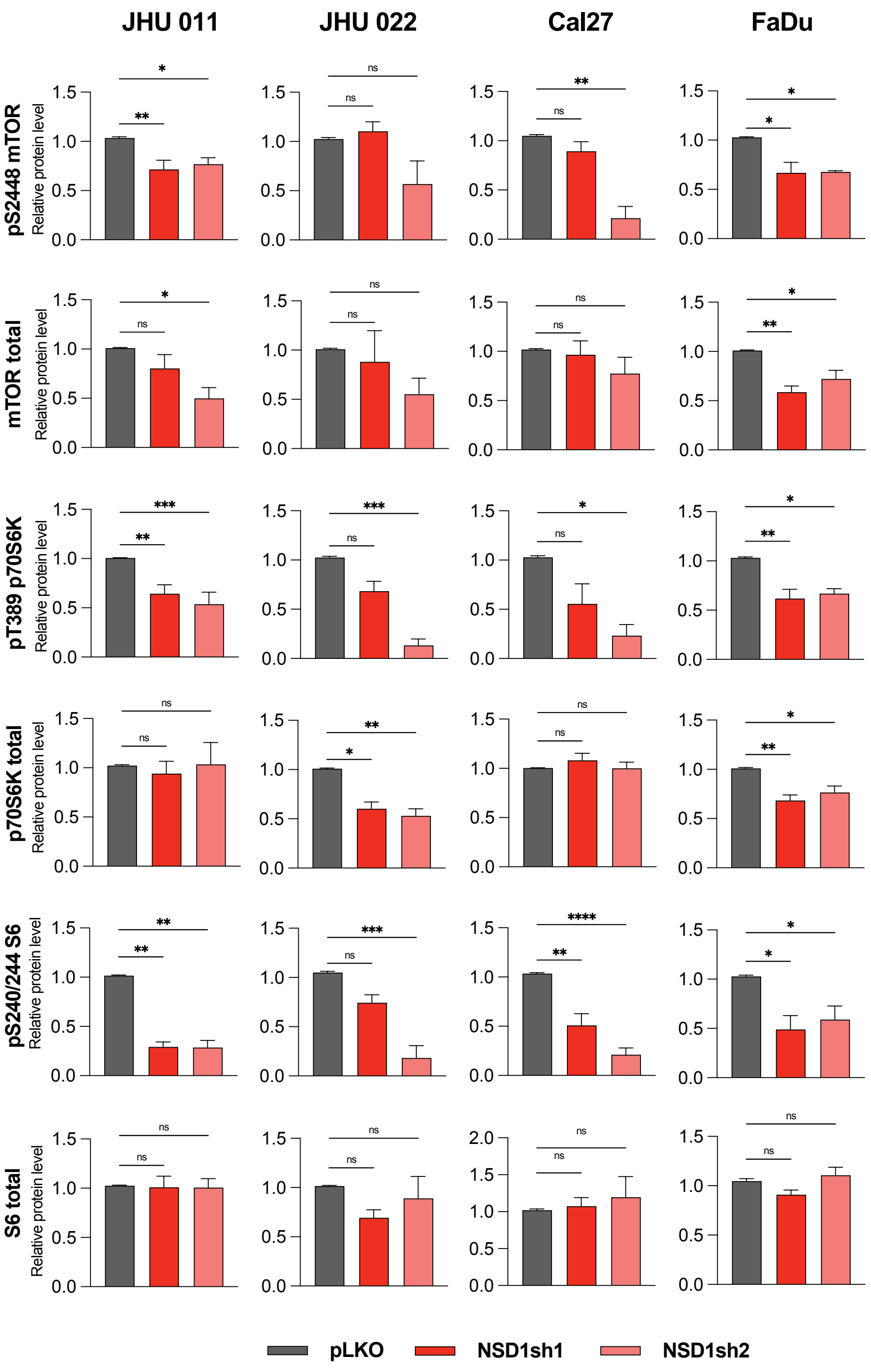

Supplementary Figure S5

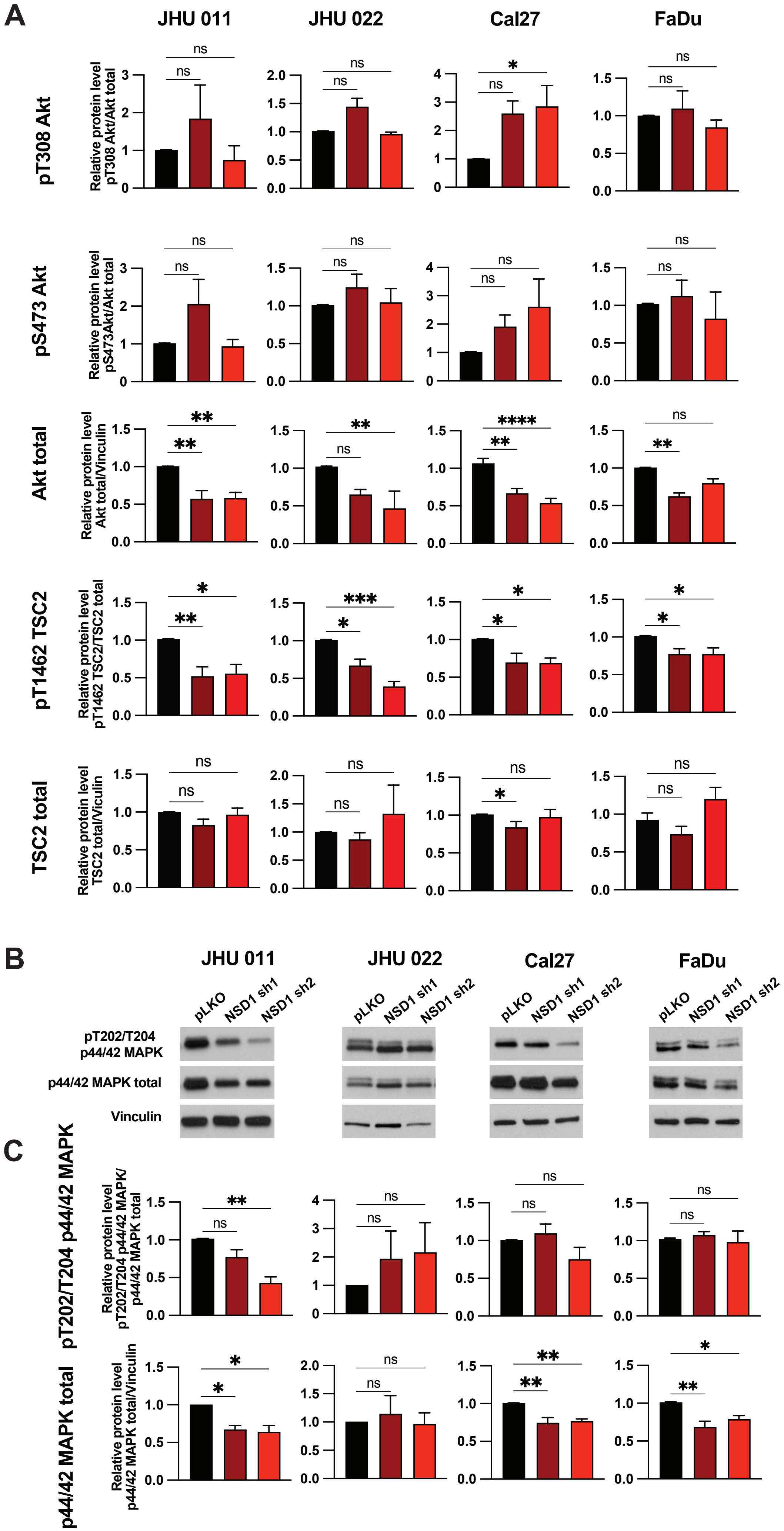

Supplementary Figure S6

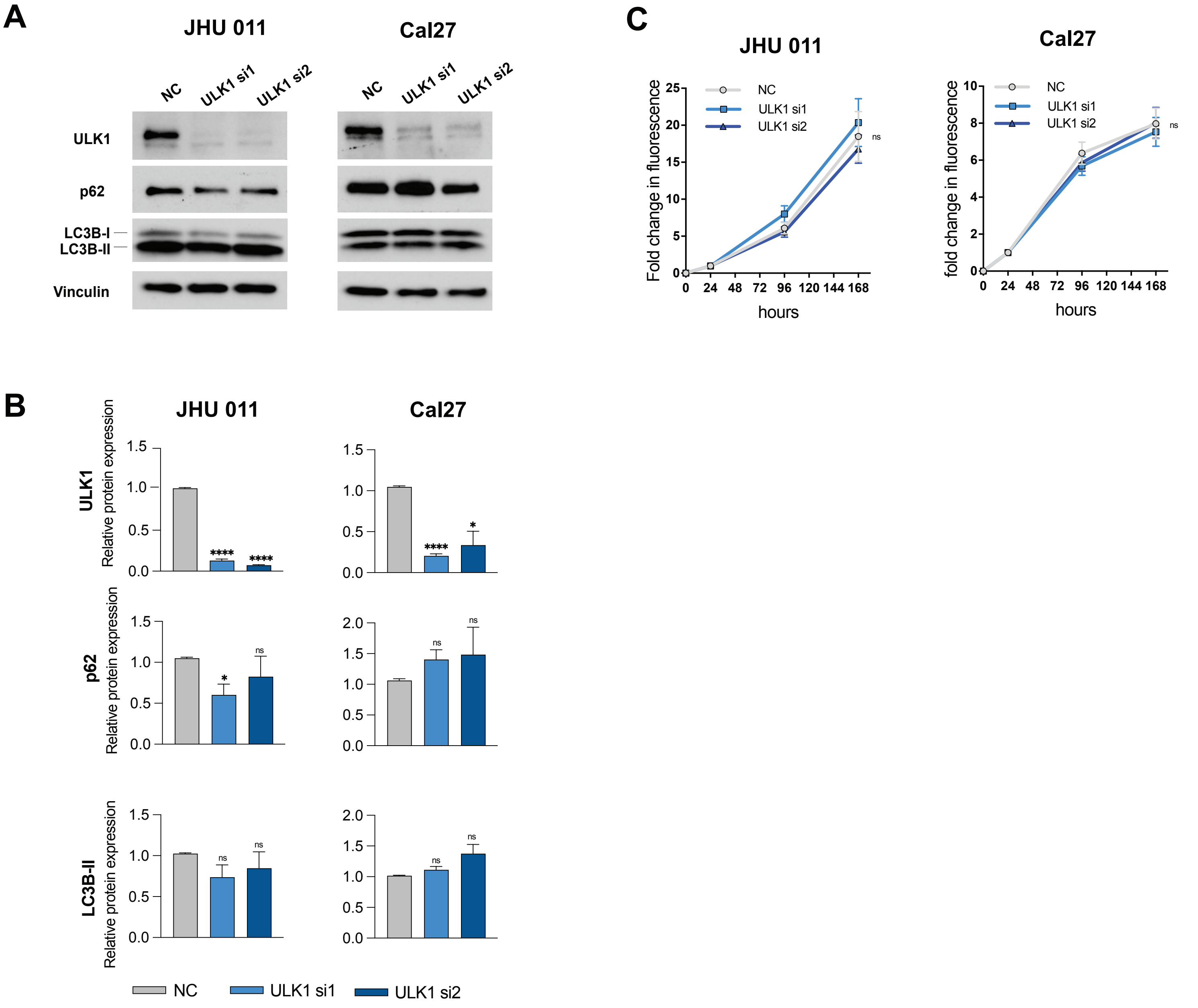

Supplementary Figure S7

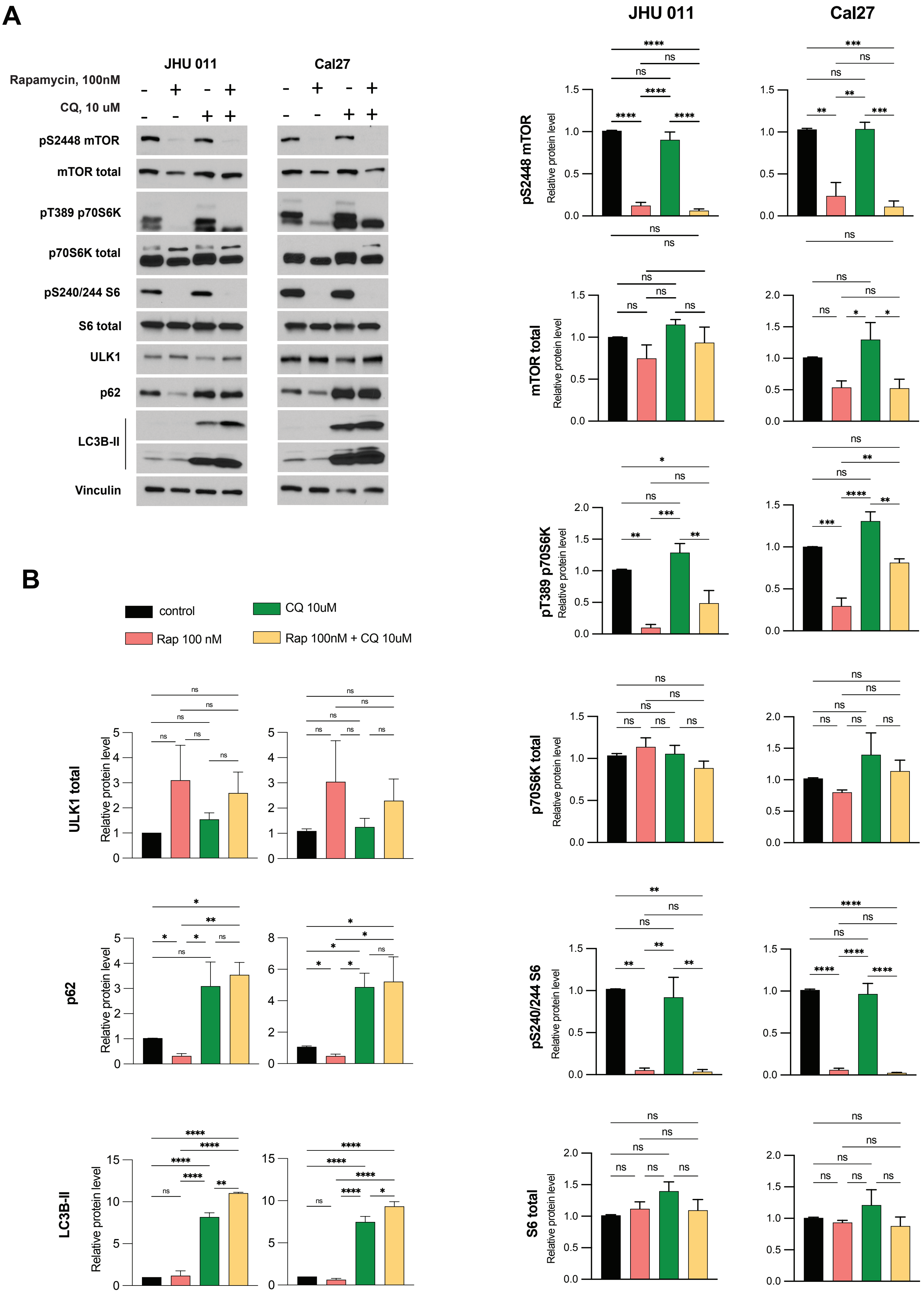

Supplement: Supplementary file 10 — Supplementary Figures [file 41420_2024_1842_MOESM10_ESM.pdf]
