# Supplementary figures and images for: NSD1 supports cell growth and regulates autophagy in HPV-negative head and neck squamous cell carcinoma
Source: Cell Death Discov. 2024 Feb 13;10:75. doi: 10.1038/s41420-024-01842-6 (PMC10861597; doi:10.1038/s41420-024-01842-6)

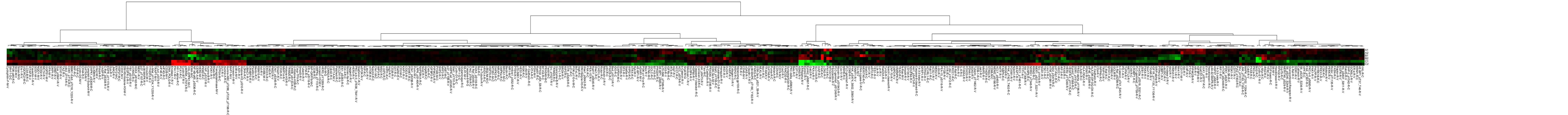

## Data Distribution

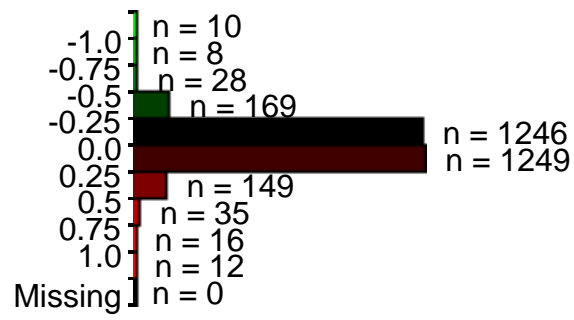

Supplement: Supplementary file 11 — Supplementary Data Set 1 [file 41420_2024_1842_MOESM11_ESM.zip › Supplementary Data Set 1/RPPA_Cal27_heatmap.pdf]

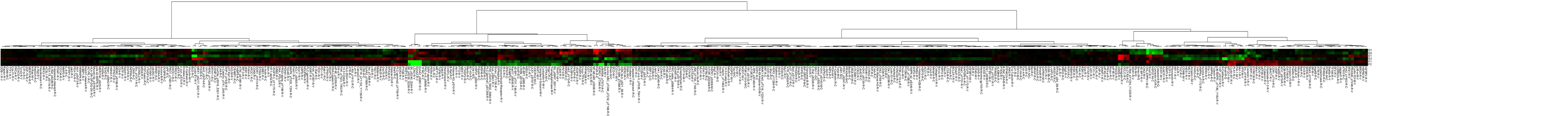

Data Distribution

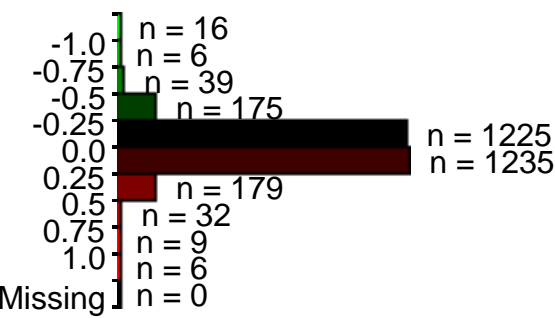

Supplement: Supplementary file 11 — Supplementary Data Set 1 [file 41420_2024_1842_MOESM11_ESM.zip › Supplementary Data Set 1/RPPA_JHU 011_heatmap.pdf]
